# Facile high-throughput forward chemical genetic screening by *in situ* monitoring of glucuronidase-based reporter gene expression in *Arabidopsis thaliana*

**DOI:** 10.3389/fpls.2015.00013

**Published:** 2015-01-29

**Authors:** Vivek Halder, Erich Kombrink

**Affiliations:** Chemical Biology Laboratory, Max Planck Institute for Plant Breeding ResearchCologne, Germany

**Keywords:** chemical screening, chemical genetics, high-throughput screening, bioactive small molecules, β-glucuronidase activity, reporter gene expression, salicylic acid

## Abstract

The use of biologically active small molecules to perturb biological functions holds enormous potential for investigating complex signaling networks. However, in contrast to animal systems, the search for and application of chemical tools for basic discovery in the plant sciences, generally referred to as “chemical genetics,” has only recently gained momentum. In addition to cultured cells, the well-characterized, small-sized model plant *Arabidopsis thaliana* is suitable for cultivation in microplates, which allows employing diverse cell- or phenotype-based chemical screens. In such screens, a chemical's bioactivity is typically assessed either through scoring its impact on morphological traits or quantifying molecular attributes such as enzyme or reporter activities. Here, we describe a facile forward chemical screening methodology for intact *Arabidopsis* seedlings harboring the β-glucuronidase (GUS) reporter by directly quantifying GUS activity *in situ* with 4-methylumbelliferyl-β-D-glucuronide (4-MUG) as substrate. The quantitative nature of this screening assay has an obvious advantage over the also convenient histochemical GUS staining method, as it allows application of statistical procedures and unbiased hit selection based on threshold values as well as distinction between compounds with strong or weak bioactivity. At the same time, the *in situ* bioassay is very convenient requiring less effort and time for sample handling in comparison to the conventional quantitative *in vitro* GUS assay using 4-MUG, as validated with several *Arabidopsis* lines harboring different GUS reporter constructs. To demonstrate that the developed assays is particularly suitable for large-scale screening projects, we performed a pilot screen for chemical activators or inhibitors of salicylic acid-mediated defense signaling using the *Arabidopsis PR1p::GUS* line. Importantly, the screening methodology provided here can be adopted for any inducible GUS reporter line.

## Introduction

In search for new tools that aid the dissection of complex biological processes, chemical genetics has been recognized as alternative experimental strategy to classical genetics approaches. Its strength lies in the potential to circumvent problems that are commonly encountered in classical genetics, such as redundancy, lethality, or pleiotropy of gene functions (Blackwell and Zhao, 2003; Stockwell, 2004; Hicks and Raikhel, 2012). For example, small molecules can in principle target multiple members of a protein family or, alternatively, the effects they exert can be temporally controlled and possibly reversed by withdrawing the chemical from the system. However, in contrast to animal systems, which are nurtured from drug discovery programs and cancer research, the application of chemical genetics in basic plant research stands quite in contrast to industrial applications such as pesticide (herbicide and fungicide) discovery and has only recently found broader application as documented in a number of reviews (Blackwell and Zhao, 2003; Raikhel and Pirrung, 2005; Kaschani and van der Hoorn, 2007; Hicks and Raikhel, 2009, 2012, 2014; Tóth and van der Hoorn, 2010).

Fundamentally, the key similar feature between chemical genetics and classical genetics is the generation of recognizable phenotypes at the whole plant, organ, cell, or subcellular level. While in genetic approaches phenotypes are created by mutations that result in altered protein expression or function, chemicals mostly interfere with protein functions directly, but when this alteration affects transcription factors or upstream components it may also result in modified gene expression. Correspondingly, numerous screenable phenotypes can be used for chemical interference and the model plant *Arabidopsis thaliana* is particularly suitable for such approaches. This is not only because of its small size, permitting easy cultivation in 96-well microplate format either on agar or in liquid medium, but also because large collections of mutants and transgenic lines are available, allowing to perform a diversity of phenotypic and reporter-based chemical screening strategies. Likewise, cultured cells are a prime choice for chemical screens. However, screening at the whole plant level offers its own advantages to monitor morphological responses that are dependent on multicellular structures such as root growth, cell-wall formation, seed germination, hypocotyl elongation and other developmental processes, as well as organ- and cell-type-specific gene expression *via* selective reporter readouts. In recent years, numerous chemical screens covering many areas of plant biology have demonstrated the increasing impact of chemical genetics on basic plant research, including some impressive success stories in which for selected small molecules the cognate targets have been identified (Hicks and Raikhel, 2014). There are multiple examples addressing questions related to plant hormone signaling, i.e., responses to auxin, abscisic acid (ABA), jasmonic acid (JA), or brassinosteroids (Hayashi et al., 2003, 2008; Zhao et al., 2003; Armstrong et al., 2004; Walsh et al., 2006; Gendron et al., 2008; De Rybel et al., 2009; Park et al., 2009; Meesters et al., 2014), endomembrane trafficking (Zouhar et al., 2004; Surpin et al., 2005; DeBolt et al., 2007; Rojas-Pierce et al., 2007; Kim et al., 2010), plant pathogen interactions and plant immune responses (Serrano et al., 2007, 2010; Schreiber et al., 2008; Knoth et al., 2009; Noutoshi et al., 2012), and cellulose biosynthesis resp. cell wall formation (Desprez et al., 2002; Yoneda et al., 2007; Park et al., 2014). However, the most impressive example of groundbreaking work with small molecules was the identification and use of a novel ABA agonist, pyrabactin, that led to the identification of the long-searched-for ABA receptor (Melcher et al., 2009; Park et al., 2009; Santiago et al., 2009; Cutler et al., 2010).

In plant chemical genetic screens, the GUS reporter system has frequently been used. The simplicity and easiness of the histochemical GUS staining method, which relies on cleavage of 5-bromo-4-chloro-3-indolyl-β-D-glucuronide (X-Gluc) and formation of a blue-colored precipitate, made this approach a suitable and preferred choice for monitoring activity (phenotypic evaluation) in large-scale chemical screening approaches (Hayashi et al., 2003; Armstrong et al., 2004; Serrano et al., 2007; Gendron et al., 2008; Knoth et al., 2009). However, on the down side, this method provides only qualitative data, which are prone to subjective decisions and biased hit selection. Alternatively, GUS activity can be quantitatively determined by spectrophotometrical or fluorimetrical assays monitoring the cleavage of p-nitrophenyl-β-D-glucuronide or 4-methylumbelliferyl-β-D-glucuronide (4-MUG), respectively (Jefferson et al., 1987). Although reliable and robust, the shortcomings of these assays are that they are labor-intensive and time-consuming, as they require tissue homogenization and protein extraction, which renders these assays unsuitable for screening of large libraries. Alternatively, luciferase- or GFP-based reporter systems, allowing monitoring of true *in vivo* activities, are also suitable for chemical screening, but as these systems are less abundant than GUS-based reporters, there are only few documented applications (Yoneda et al., 2007; Tóth et al., 2012; Forde et al., 2013; Motte et al., 2013; Meesters et al., 2014).

Since GUS is the prevailing reporter system in plants, we wanted to combine the best out of both outlined approaches of GUS activity determination for a screening platform, and thus we explored whether the ease of the histochemical GUS staining method could be merged with the advantages of quantitative enzyme assays. To this end, we have established a simple chemical screening methodology, which is based on detergent-facilitated infusion of 4-MUG substrate through any GUS expressing plant tissue and direct quantification of fluorescence emitted by the released 4-methylumbelliferone (4-MU) in the same solution (Blázquez, 2007). Importantly, this assay is not only fast, robust and reliable, but also provides quantitative (or semi-quantitative) data directly *in situ*, thereby minimizing sample handling and allowing unbiased identification of hits *via* numeric threshold values derived from statistical procedures (Malo et al., 2006; Birmingham et al., 2009). To demonstrate the potential and superiority of our screening methodology, we used the transgenic *A. thaliana* line harboring the salicylic acid (SA)-responsive *PR1p::GUS* reporter to screen separately for both activators and inhibitors of SA signaling. *PATHOGENESIS-RELATED 1* (*PR1*) is as a canonical SA marker gene, regulated by multiple transcription factors, such as TGAs and WRKYs, and it is robustly up-regulated upon plant infection with biotrophic pathogens and during the systemic immune response (Vlot et al., 2009; Tsuda et al., 2013). In this small pilot experiment, we faithfully identified the known strong activator acetylsalicylic acid (ASA) and the translation inhibitor cycloheximide (CHX), but additional modulators of *PR1* gene expression that exert only weak effects were also captured. Thus, as expected from a quantitative assay, our method enables facile, automatic data acquisition and can also reliably distinguish between compounds with high and low potency. With this facile method at hand, large-scale screening campaigns using any GUS-expressing *Arabidopsis* line can be carried out in a time-, labor-, and cost-effective manner.

## Materials and methods

### Plant material and growth conditions

In this study we used *A. thaliana* Columbia-0 (Col-0) transgenic lines carrying the following reporter genes in the Col-0 (or Col-5) genomic background: *PR1p::GUS* (Shapiro and Zhang, 2001), *DR5::GUS* (Ulmasov et al., 1997), *WRKY29p::GUS* (Serrano et al., 2007), and *DC3::GUS* (Chak et al., 2000). *Arabidopsis* seeds were surface-sterilized and seedlings grown hydroponically in 96-well microplates (PerkinElmer Inc., Germany) containing 0.2 ml of half-strength MS basal salt medium (Murashige and Skoog, 1962) supplemented with 0.5% sucrose. After stratification for 2 days at 4°C in the dark, plates were placed for 12 days in a growth chamber at a day/night cycle of 16/8 h at 21/19°C, respectively.

### Analysis of gene expression in GUS reporter lines

Gene expression of β-glucuronidase (GUS) reporter lines was induced by treatment with the appropriate phytohormones as previously reported to yield maximum activity, i.e., *PR1p::GUS* was treated with 200 μM SA for 24 h, *DR5p::GUS* with 5 μM indole 3-acetic acid (IAA) for 4 h, *DC3p::GUS* with 100 μM ABA for 24 h, and *WRKY29p::GUS* with 1 μM peptide epitope of bacterial flagellin (flg22) for 4 h. Following this treatment, the medium was removed by aspiration and seedlings were used immediately (or stored at −80°C) for quantification of GUS activity by *in situ* or *in vitro* assays. To reveal the organ- and cell-type-specific expression patterns of reporter genes, histochemical GUS staining was performed with the chromogenic substrate 5-bromo-4-chloro-3-indolyl-β-D-glucuronide (X-gluc) as previously described (Ancillo et al., 2003) using 12-day-old seedlings after treatment as specified above.

#### Quantification of GUS activity *in vitro*

The quantitative GUS assay was carried out as previously described (Sprenger-Haussels and Weisshaar, 2000). In brief, tissue samples (1–4 seedlings corresponding to 20–100 mg) were transferred to microtubes, homogenized in extraction buffer (100 mM potassium phosphate, 1 mM DTT, pH 7.5) and debris removed by centrifugation (30 min, 13,000 g, 4°C). The clear supernatant (50 μL) was mixed with GUS assay buffer (50 μL) containing 2 mM 4-methylumbelliferyl-β-D-glucuronide (4-MUG), 50 mM Na-phosphate pH 7.0, 1 mM EDTA, 0.1% Triton X-100, 10 mM β-mercaptoethanol. Aliquots (20 μL) were sampled after 0, 30, and 60 min incubation at 37°C (unless otherwise stated), mixed with 0.2 mL 0.2 M Na_2_CO_3_ and 4-MU fluorescence was determined in a microplate reader (FluoroCount, Packard Bioscience, Meriden, Connecticut) using an excitation/emission wavelength of 365/455 nm. GUS activity was calculated using the ΔE_455_ increments (0–30 and 30–60 min) and appropriate 4-MU standards (50–5000 pmol). Specific activities were related to the protein concentration determined according to Bradford (Bradford, 1976) with bovine serum albumin as standard. All reported values are the mean (±SD) of at least four biological replicates.

#### Quantification of GUS activity in intact seedlings (*in situ*)

To adjust the quantitative GUS assay for large-scale screening applications, we optimized a previously reported method (Blázquez, 2007) by minimizing handling time and effort. In brief, single 12-day-old seedlings grown in 96-well microplates were incubated with 150 μL lysis buffer (50 mM sodium phosphate, pH 7.0, 10 mM EDTA, 0.1% Triton X-100) containing 1 mM 4-MUG at 37°C for 90 min, unless otherwise stated. Of note, seedlings should be completely submerged in lysis buffer to allow ubiquitous substrate supply. At the end of the incubation period, 50 μL 1 M Na_2_CO_3_ (stop solution) was added to each well and 4-MU fluorescence directly determined in a microplate reader as before (excitation/emission wavelength of 365/455 nm). Activity is either directly expressed as relative light units (RLU per assay or seedling) or was converted to molar units using a standard curve (150 μL 50–1000 μM 4-MU in lysis buffer, plus 50 μL stop solution). All results are typically the mean (±SD) of at least four biological replicates.

### Chemical library screening

**Figure 1:**
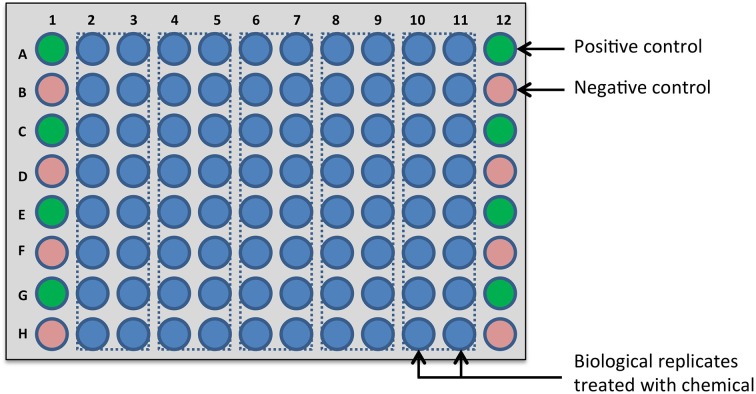
**Design of chemical screening plate**. In the screens described here, each chemical is tested in two biological replicates in the central wells of a 96-well microplate (blue circles), allowing 40 chemicals to be analyzed. This design is recommended, because in commercial compound libraries, 80 different compounds are generally stored in the middle of 96-well plates and the first and last columns are left empty. Correspondingly, column 1 and column 12 are available for controls and to minimize edge-related bias, the eight positive controls (green circles) and the eight negative controls (red circles) are distributed across these columns in alternating order.

A small compound library, comprising 40 hand-picked chemicals (1 mM dissolved in DMSO), was used for screening. *Arabidopsis* seedlings harboring the *PR1p::GUS* reporter were grown in 96-well microplates for 12 days and before chemical treatment, growth medium was removed and replaced by fresh half-strength MS medium. To conditionally modulate SA signaling, seedlings were pretreated with chemicals (dissolved in DMSO) at a final concentration of 20 μM for 1 h before addition of 200 μM SA (dissolved in DMSO) to induce *PR1p::GUS* expression and subsequent incubation for 24 h unless otherwise stated (screening for inhibitors). Alternatively, omission of SA allowed screening for activators of *PR1p::GUS* expression. All chemicals were analyzed in two replicates and their activity normalized to control samples (without added chemical) that were contained on the same microplate (first and last column). The organization of samples in 96-well microplates is shown in Figure 1.

### Statistical analysis

The quantitative data analysis was performed in Excel spreadsheets with the embedded basic statistical functions (mean, standard deviation, Student's *t*-test, r.m.s. linear regression).

A common quality metric for evaluation and validation of high-throughput screening assays are the *Z* and *Z*' factors (Zhang et al., 1999; Birmingham et al., 2009). The *Z*' factor, often used during assay optimization, relies on high-value (positive) and low-value (negative) controls and is calculated by Equation (1), with μ representing the mean and σ the standard deviation of the high-value (subscript “hc”) and low-value (subscript “lc”) controls, respectively.

(1) $$Z^{\prime }\text{ factor}=1-\frac{\left(3\sigma_{hc}+3\sigma_{lc}\right)}{\left|\mu_{hc}-\mu_{lc}\right|}$$

The *Z*' factor ranges from negative infinity to 1, with values >0.5 indicating an excellent assay, >0 an acceptable assays and <0 an unacceptable assay. Correspondingly, the *Z* factor may be calculated using actual screening data (high values) instead of separate positive control values and thus serves to directly assess performance of the screen (Zhang et al., 1999; Birmingham et al., 2009).

The *Z* score, not to be confused with the *Z* and *Z*' factors, representing the number of standard deviations from the mean, is frequently used to normalize screening data such that individual measurements are rescaled relative to the whole-plate variation (Malo et al., 2006; Birmingham et al., 2009). The *Z* score was calculated by Equation (2), with *x_i_* representing the raw value of the individual compound *i*, μ and σ are the mean and standard deviation, respectively, of all values within a plate.

(2) $$Z\text{ score}=\frac{x_{i}-\mu }{\sigma }$$

## Results

### Direct quantification of GUS activity in intact *Arabidopsis* seedlings

**Figure 2:**
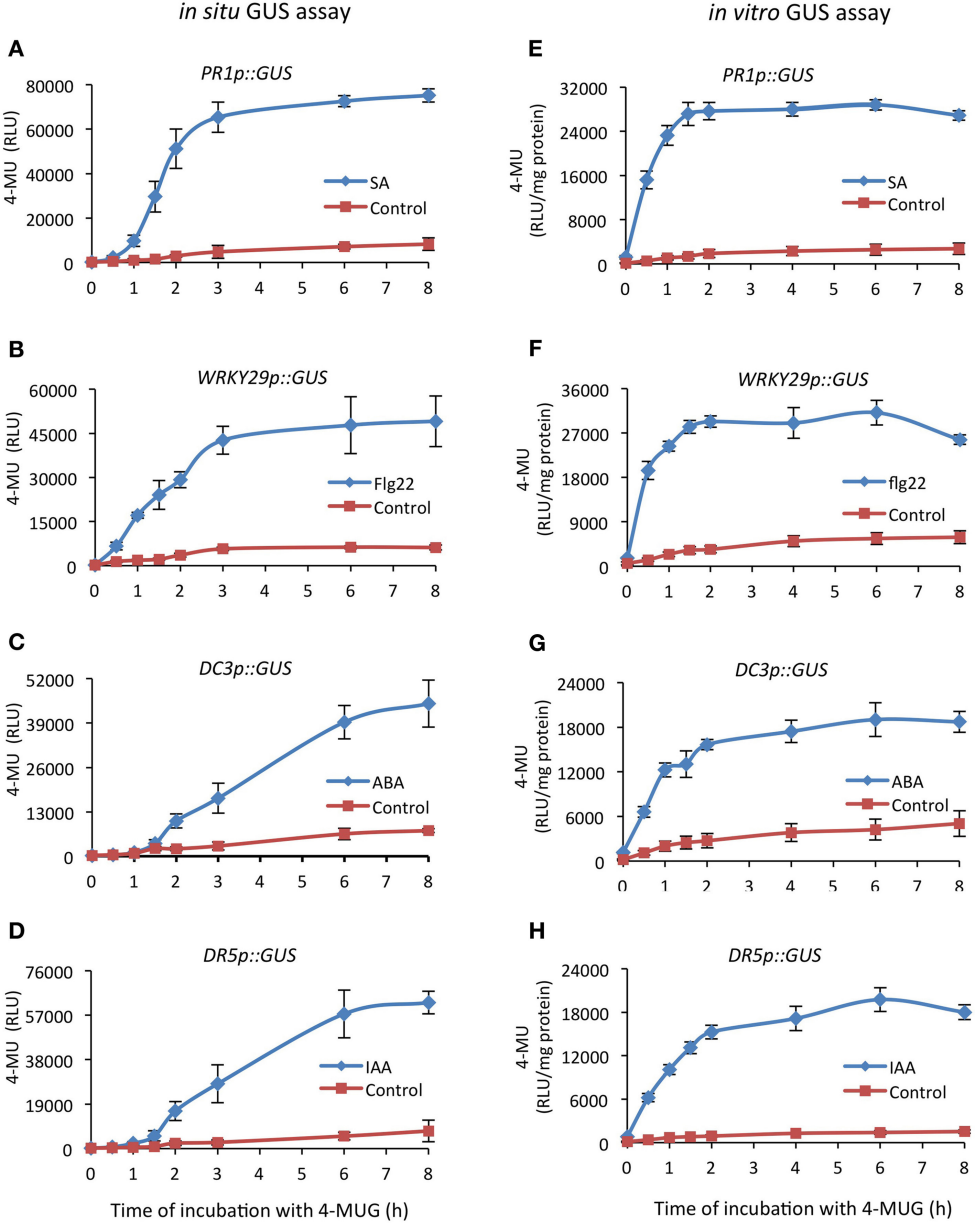
**Comparison of GUS activity determined in whole seedlings (*in situ*) and in protein extracts (*in vitro*)**. Seedlings of transgenic *Arabidopsis thaliana* lines harboring different inducible promoter–GUS fusions were grown for 12 days hydroponically in microplates and then treated with the respective inducer (or solvent as control) for an appropriate time period to obtain high expression levels of the reporters. **(A,E)** *PR1p::GUS* seedlings received 200 μM SA for 24 h, **(B,F)** *WRKY29p::GUS* received 1 μM flg22 for 4 h, **(C,G)** *DC3p::GUS* received 100 μM ABA for 24 h and **(D,H)** *DR5p::GUS* received 5 μM IAA for 4 h. Following this treatment, the medium was removed and for monitoring GUS activity *in situ* **(A–D)**, seedlings were incubated with the substrate 4-MUG (1 mM) for the indicated time periods before the reaction was terminated by addition of stop solution (Na_2_CO_3_). The released reaction product, 4-MU, was directly quantified by its fluorescence in a microplate reader. For quantifying GUS activity *in vitro* **(E–H)**, seedlings were homogenized and conversion of the substrate 4-MUG (2 mM) in clarified protein extracts was determined as described in the Materials and Methods Section. 4-MU release is given in relative light units (RLU) emitted from the whole *in situ* assay **(A–D)** or normalized to the protein concentration for the *in vitro* assay **(E–H)**. All values represent the mean (±SD) of four biological replicates.

We wanted to establish a facile GUS assay that does not require tissue homogenization and yet provides a reliable, quantitative output that is suitable for large-scale chemical library screening. Therefore, we used *Arabidopsis* seedlings harboring different inducible GUS reporter constructs, which were grown hydroponically in 96-well microplates and treated accordingly to provide high GUS activity. Such seedlings were then directly incubated with GUS assay buffer, which was supplemented with Triton X-100 to enhance the permeability of both the substrate 4-MUG and the product 4-MU throughout the tissue. The release of the product (4-MU, monitored by its fluorescence) occurred with a delay of 20–60 min, followed by a linear increase for about 2 h until the substrate was depleted (Figure 2). Apparently, the delay of product release is inversely correlated with total GUS activity; strong promoters, such as *PR1* or *WRKY29* (Figures 2A,B), providing high levels of expression (and enzyme activity) showed shorter delays of substrate release in comparison to DC3 or DR5 (Figures 2C,D), which yield lower expression levels and extended delays.

**Figure 3:**
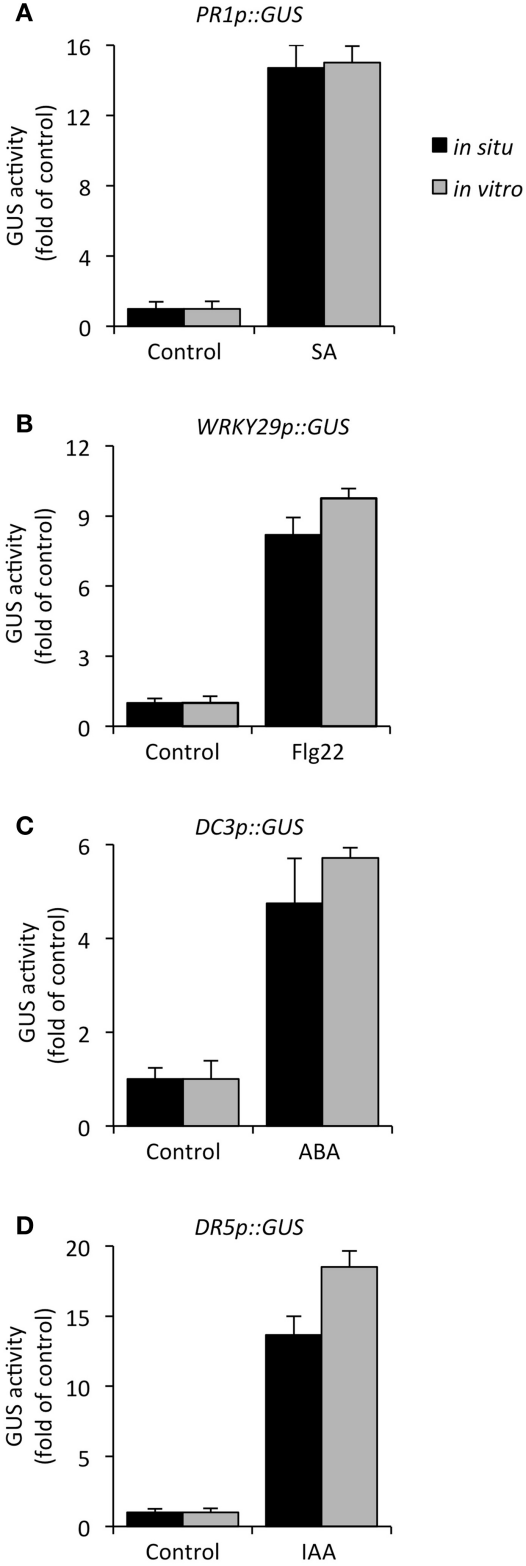
**Comparison of induced expression of diverse promoter–GUS reporter genes as determined by *in situ* and *in vitro* GUS assays**. Twelve-day-old transgenic *Arabidopsis* seedlings were appropriately treated to obtain high reporter gene expression: **(A)** *PR1p::GUS* (200 μM SA, 24 h), **(B)** *WRKY29p::GUS* (1 μM flg22, 4 h), **(C)** *DC3p::GUS* (100 μM ABA, 24 h), and **(D)** *DR5p::GUS* (5 μM IAA, 4 h). GUS activity *in situ* (black bars) was determined after incubation of whole seedlings with the substrate 4-MUG (1 mM) for 2 h and it is compared to GUS activity (initial rate) determined *in vitro* (gray bars) using protein extracts prepared from seedlings that were treated identically. For better comparison, the resulting activities *in situ* [relative light units (RLU) per assay] and *in vitro* (pmol min^−1^ mg^−1^ protein) are normalized to untreated control samples, thus showing fold of induction in response to treatment. All values represent the mean (±SD) of four biological replicates.

To confirm that the *in situ* GUS assay faithfully records activity, we also determined rates of substrate conversion *in vitro* by a conventional GUS activity assay (Sprenger-Haussels and Weisshaar, 2000), using seedlings that were subjected to the same treatments. As expected, in protein extracts the release of the product (4-MU) occurred instantaneously but otherwise followed a similar time course, as in intact seedlings (Figures 2E–H). Next, we directly compared the specific GUS activity profiles in biological samples, i.e., transgenic *Arabidopsis* lines harboring different reporter constructs, that were treated accordingly to provide high expression levels of the respective reporter gene. As apparent from Figure 3, our *in situ* method and the established *in vitro* GUS assay generally recorded nearly identical induction of activity in response to specific treatments in all tested reporter lines, ranging between 15-fold for *PR1p::GUS* (SA responsive) and 5-fold for *DC3p::GUS* (ABA responsive) when comparing positive and negative controls. Of note, the *in situ* GUS activity in this experiment was determined from a fixed incubation period of 2 h for all samples, whereas the *in vitro* activity assay recorded initial rates over maximally 1 h (cf Figure 2). Therefore, as result of delayed substrate release, the *in situ* method had a tendency to provide lower values, ranging from a maximum deviation of -30% (*DR5p::GUS*, Figure 3D) to virtually identical values (*PR1p::GUS*, Figure 3A). From this we conclude that GUS activity can be directly and reliably estimated in intact seedlings, but the conditions need to be adjusted to each particular reporter lines such that product release remains in the linear range (or near linear range) and not all 4-MUG has been consumed. For the *PR1p::GUS* line, we selected an incubation time of 90 min for all subsequent experiments (cf Figure 2A).

### Robust and reliable GUS quantification in fresh and frozen *Arabidopsis* seedlings

**Figure 4:**
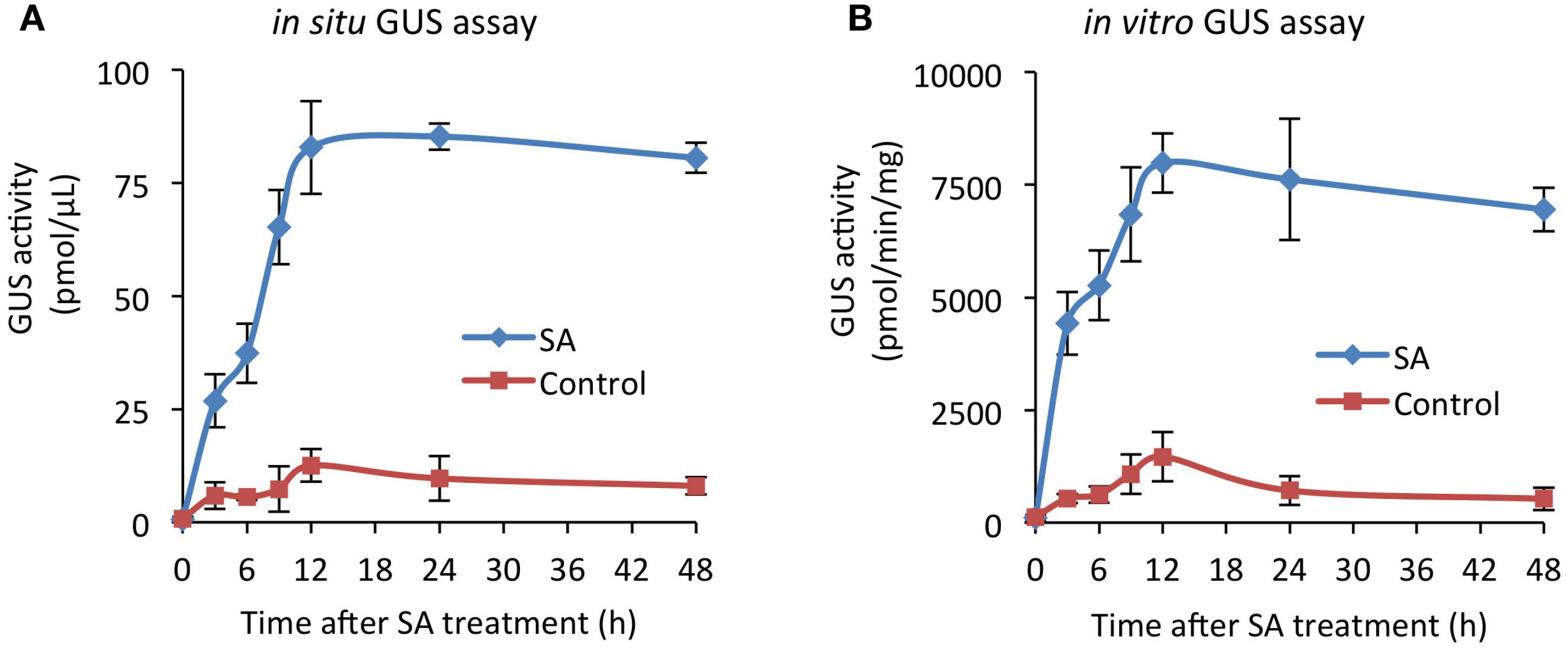
**Time course of *PR1p::GUS* expression upon treatment with SA**. *Arabidopsis* seedlings harboring the SA-responsive *PR1p::GUS* reporter gene, grown for 12 days in liquid culture, were treated with 200 μM SA (or 0.2% DMSO as control) for the indicated time periods. **(A)** GUS activity was determined with intact seedlings (*in situ*) and **(B)** in total extracts (*in vitro*) derived from seedlings of the same experiment. Specific activities are derived from 4-MU standard curves and are normalized to assay volume **(A)** or total extractable protein **(B)**. All values represent the mean (±SD) of four biological replicates.

To further validate the reliability and robustness of GUS activity quantification in intact seedlings, we applied the *in situ* GUS assay to analyze the time course of *PR1p::GUS* expression upon treatment with SA. Here, a standard curve with known 4-MU concentrations was used to normalize the activity, i.e., the emitted fluorescence, which was again compared to the GUS activity determined *in vitro*. As shown in Figure 4, both assays provide a similar result (i.e., GUS activity profiles), demonstrating that *PR1* gene expression is rapidly up-regulated, reaching a maximum at 12 h and slowly declining thereafter. In control seedlings, treated with solvent (DMSO), only a low activity increase occurred.

**Figure 5:**
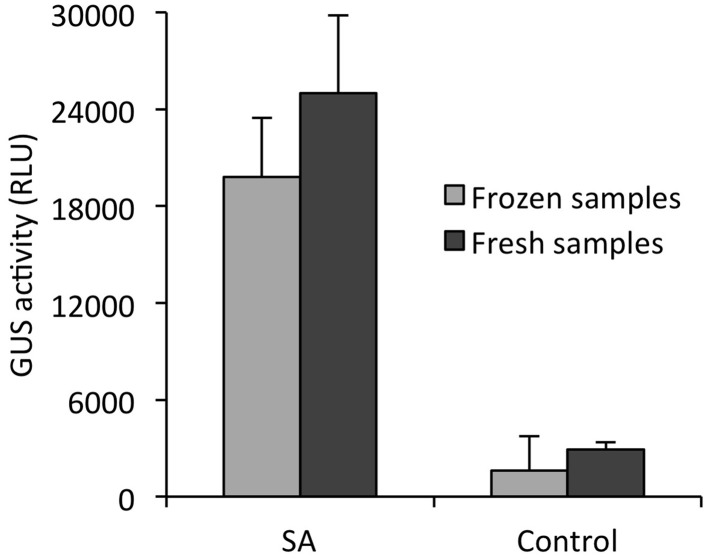
**Comparison of *in situ* GUS activity in fresh and frozen seedlings**. *Arabidopsis* seedlings harboring the SA-responsive *PR1p::GUS* reporter gene, grown for 12 days in liquid culture, were treated with 200 μM SA or 0.2% DMSO (control) for 24 h. Half of the samples served for instant determination of GUS activity *in situ* (black bars) as described in Materials and Methods. The other half was frozen and stored at −80°C for 4 weeks. For determining GUS activity, frozen seedlings were transferred to microplate wells prefilled with assay buffer containing 1 mM 4-MUG and incubated for 90 min before quantifying 4-MU fluorescence. Activity is given in relative light units (RLU) emitted from total assays and all values represent the mean (±SD) of four biological replicates.

For many biological applications it is necessary or useful to freeze samples for subsequent bioassays. We therefore explored whether the new GUS assay can also be performed with frozen seedlings without loss in performance. Therefore, *PR1p::GUS* seedlings were treated with SA (200 μM) as before and at the end of the incubation period (24 h) half of the samples were used to quantify GUS activity immediately. The other half was transferred to Eppendorf tubes, frozen in liquid nitrogen and stored at −80°C for 4 weeks. (Of note, for short-term storage samples can also be frozen directly in closed microplates). Without much thawing, seedlings were provided with substrate-containing lysis buffer and activity was recorded as before. The GUS activity determined in fresh and frozen seedling diverged by maximally 20% in both SA-treated and control samples (Figure 5).

We conclude, the described GUS activity assay for application with intact seedlings is robust and reliable and the facile acquisition of quantitative data makes it particularly suitable for application in large-scale screening programs.

### The GUS product 4-MU is readily released from the plant tissue

**Figure 6:**
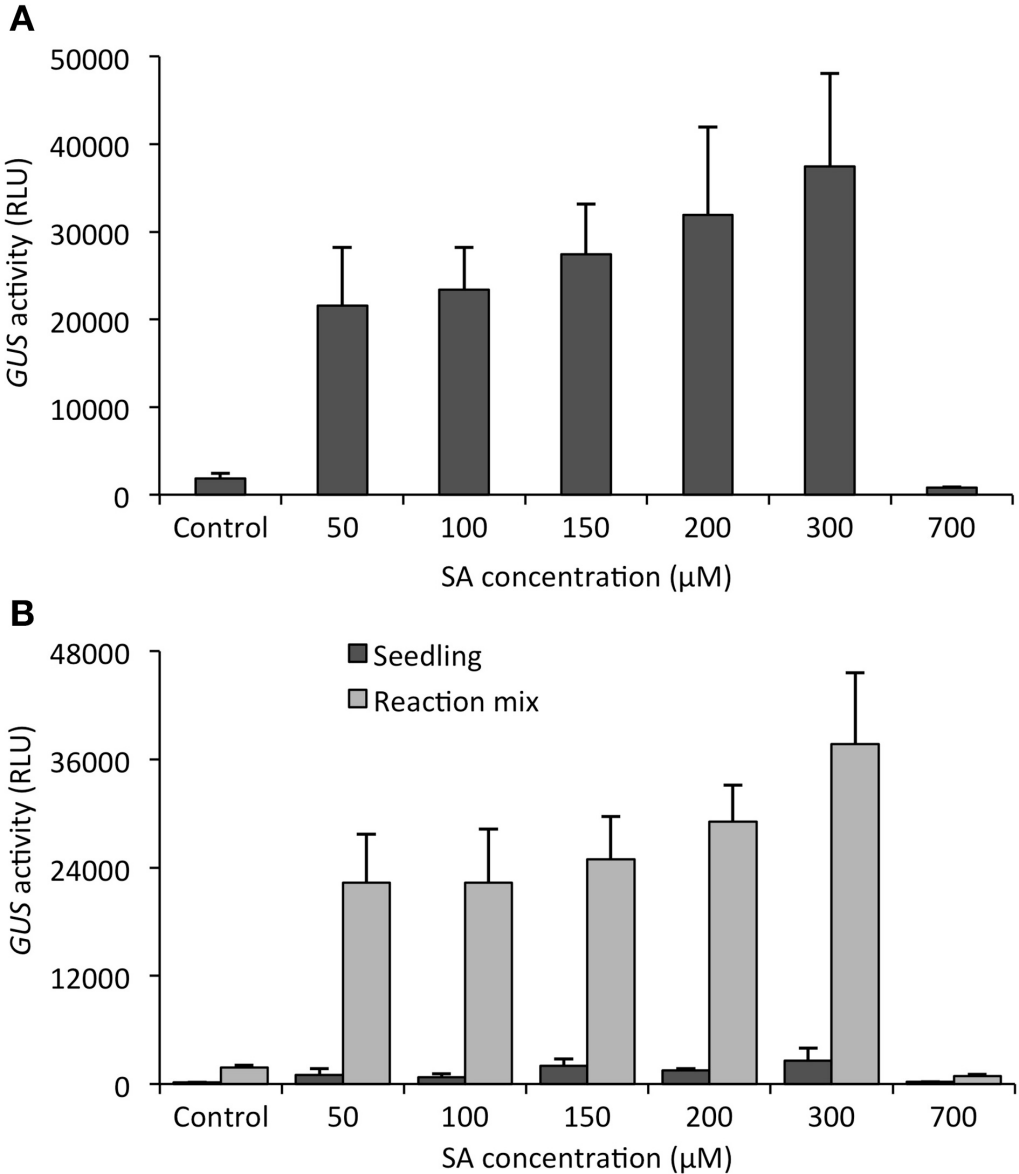
**The reaction product of the *in situ* GUS assay accumulates in the medium**. *Arabidopsis* seedlings harboring the SA-responsive *PR1p::GUS* reporter gene, grown for 12 days in liquid culture, were treated with increasing concentrations of SA (or 0.2% DMSO as control) for 24 h. **(A)** GUS activity of whole seedlings (*in situ*) was quantified as before (see Materials and Methods). **(B)** Following 4-MU quantification, seedlings were removed from first assay mixture, transferred to new microplates and 4-MU fluorescence emitted from seedlings only (black bars) or the reaction mixture devoid of seedlings (gray bars) quantified. The reaction product, 4-MU, is almost exclusively localized in the medium. All values represent the mean (±SD) of four biological replicates.

The functionality of the GUS assay with intact seedlings relies on the included detergents, Triton X-100, which facilitates penetration of substrate and product throughout the seedlings (Blázquez, 2007). To demonstrate that this is a valid assumption, we monitored whether the product of the reaction, 4-MU, indeed leaks out of the seedlings or stays within. To this end, we treated *PR1p::GUS* seedlings with various SA concentrations and after 24 h determined GUS activity (Figure 6A). From the results it is apparent that increasing SA caused higher *PR1* gene expression, reaching a maximum at 200–300 μM as previously reported (Bartsch et al., 2010). Higher SA concentrations were toxic and therefore no gene expression (GUS activity) was detectable. When from the same experiment, the seedlings were removed from the assay buffer, transferred to new microplates, and the fluorescence emanating from the seedlings and the assay buffer was separately recorded, we observed that the entire signal was almost exclusively associated with the solution (Figure 6B). This indicates that the enzyme's product, 4-MU, is readily released from the plant tissue and collected in the medium.

### Chemical library screening with GUS assay in intact seedlings

To demonstrate the general suitability of the new GUS assay methodology for chemical library screening with intact seedlings harboring inducible GUS reporter constructs, we performed a pilot screen with just 40 selected compounds, which fit in one 96-well microplate when assayed in duplicates. The general design of the screening plate, which should also be adopted for large-scale screening campaigns comprising several thousand chemicals, is shown in Figure 1; it includes positive (SA treatment) and negative (DMSO) controls alternating in the first and last column. Since we used an inducible GUS reporter system, it could be applied for bidirectional screening for either activators of gene expression or inhibitors that impair induced gene expression.

However, before proceeding directly to screening data analysis, we first assessed the quality of our assay conditions to ensure that the resulting data meet the minimum standards and permit legitimate conclusions. Therefore, we calculated the *Z*' factor, which is a common quality metric for evaluation and validation of high-throughput screening assays (Zhang et al., 1999; Birmingham et al., 2009), using the eight positive and eight negative control values included in each of the two screening plates (cf. Figure 1). The high-value (SA treated) control (RLU = 42,826 ± 5342 and 37,266 ± 2480) and low-value (DMSO treated) control (RLU = 1243 ± 459 and 2294 ± 711) represent the screening window (Supplementary Figures 1A,B) and yielded *Z*' factors of 0.58 and 0.73, respectively. By exceeding the value of 0.5, this clearly defines the SA-induced *PR1p::GUS* expression as an excellent assay for chemical screening purposes, when using the established conditions for *in situ* quantification of GUS activity.

**Figure 7:**
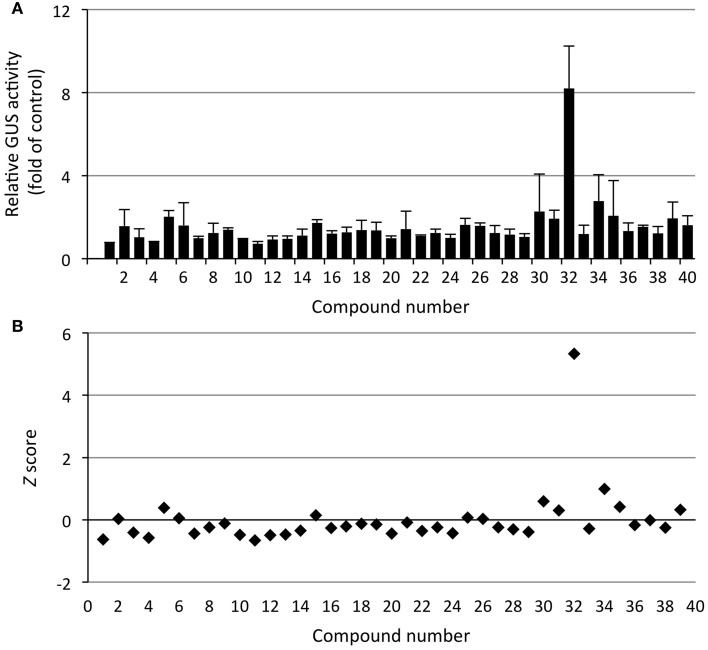
**Screening for activators of SA signaling**. *Arabidopsis* seedlings harboring the SA-responsive *PR1p::GUS* reporter gene, grown for 12 days in liquid culture, were treated with 40 diverse chemicals (20 μM) for 24 h. **(A)** GUS activity of whole seedlings (*in situ*) was quantified by incubation with 4-MUG (1 mM) for 90 min (see Materials and Methods) and normalized to the control samples (DMSO treated). Values represent the mean of duplicate samples and the error bars indicate the corresponding high and low values. One compound (**32**, acetylsalicylic acid) appeared as strong activator of reporter gene expression, causing 8-fold induction. **(B)** *Z* score transformation of the screening data (see Materials and Methods) likewise identifies compound **32** as strong hit (*Z* > 5), whereas compound **34** (cycloheximide) is a marginal hit (*Z* ≈ 1), which requires confirmation and further validation. The raw activity data of this screen are presented in Supplementary Figure 1A.

In the screen for **activators** of *PR1p::GUS* expression, 12-day-old seedlings were treated with chemicals at 20 μM for 24 h followed by instant quantification of GUS activity. Only one constituent of the library, which was identified as acetylsalicylic acid (ASA, **32)**, also named aspirin, caused an appreciable increase in GUS activity (Figure 7A). ASA has previously been demonstrated to activate plant defense responses, similar to SA (White, 1979; Spoel et al., 2003; Loake and Grant, 2007). Importantly, the recorded activity was about 8-fold higher than the negative control values (RLU = 1243 ± 459) and about 25% of the positive control values obtained with 200 μM SA (RLU = 42,826 ± 5342) (Figure 7A and Supplementary Figure 1A). To gain further confidence in our hit selection, we also calculated the *Z* score, which serves to normalize the data and also provides explicit information on the variation in sample and control measurements (Malo et al., 2006; Birmingham et al., 2009). Hit compounds are selected on the basis of a threshold value, which is typically set to a *Z* score of 2–3, i.e., SD above or below the normalized mean (*Z* score = 0). With a *Z* score > 5, ASA can be classified as strong hit, whereas weak candidates [e.g., compound **34** (cycloheximide, CHX) with a *Z score* ≈ 1] would require confirmation by additional experiments (Figure 7B).

**Figure 8:**
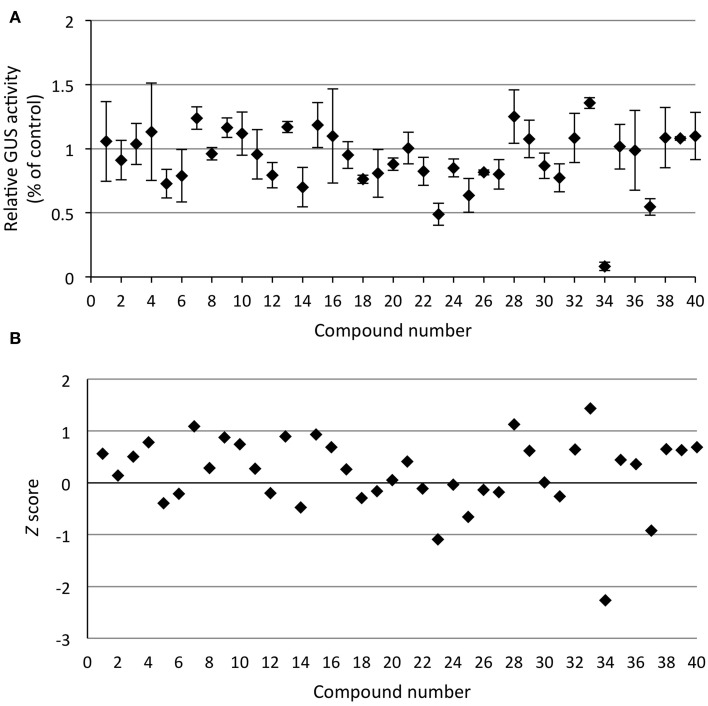
**Screening for inhibitors of SA signaling**. *Arabidopsis* seedlings harboring the SA-responsive *PR1p::GUS* reporter gene, grown for 12 days in liquid culture, were treated with 40 diverse chemicals (20 μM) for 1 h prior to addition of SA (200 μM) to induce reporter gene expression. **(A)** GUS activity of whole seedlings (*in situ*) was quantified by incubation with 4-MUG (1 mM) for 90 min (see Materials and Methods) and normalized to the SA-treated control samples. Values represent the mean of duplicate samples and the error bars indicate the corresponding high and low values. One compound (**34**, cycloheximide) appeared as strong inhibitor of reporter gene expression. **(B)** *Z* score transformation of the screening data (see Materials and Methods) likewise identifies compound **34** as strong hit (*Z* < −2), whereas compounds **23** (neosolaniol) and **37** (thiomersal) are marginal hits (*Z* ≈ −1), which require confirmation and further validation. The raw activity data of this screen are presented in Supplementary Figure 1B.

**Figure 9:**
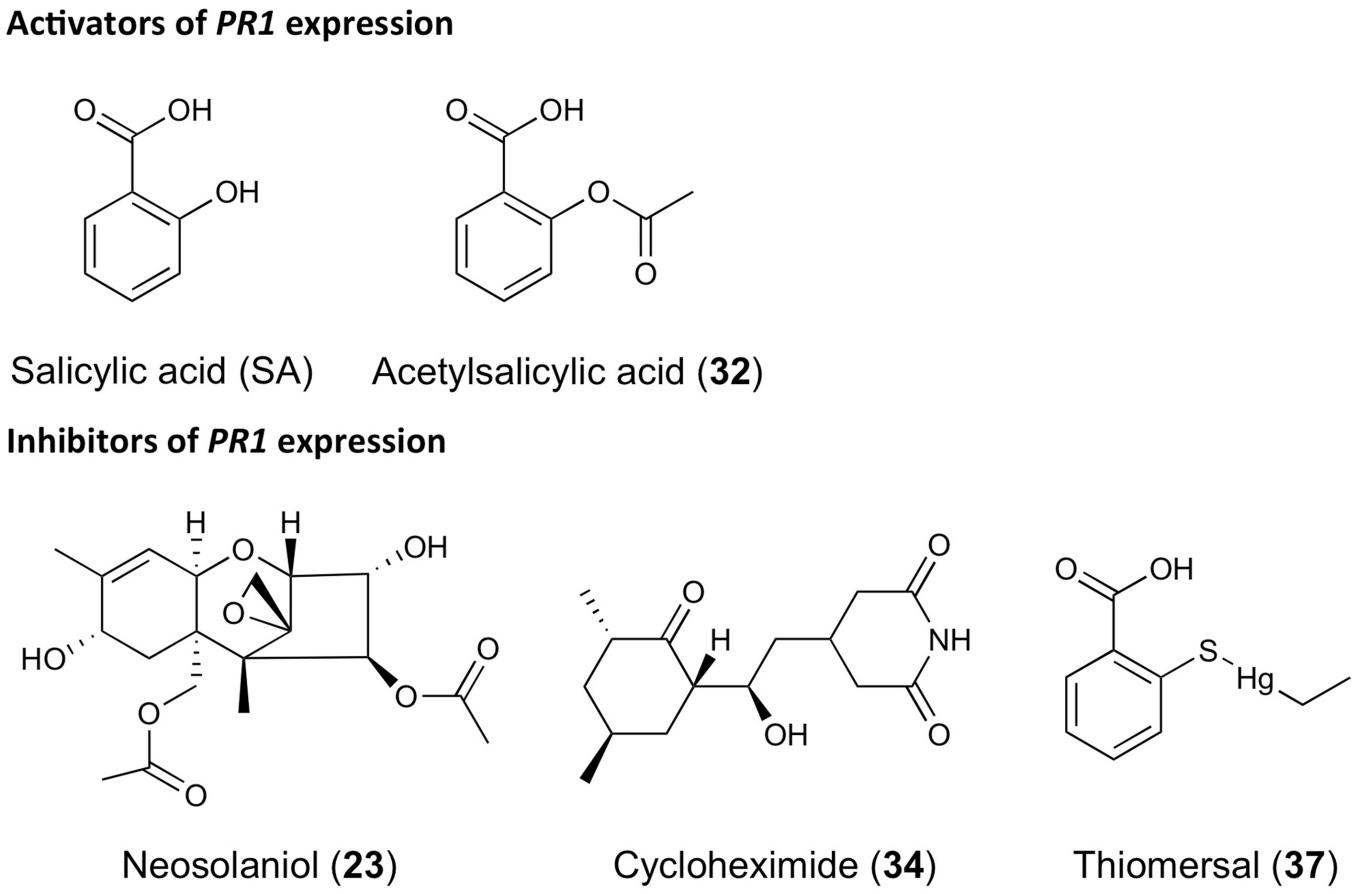
**Structures of bioactive compounds modulating *PR1* gene expression**. Examples refer to compounds mentioned in this paper that were identified in the small pilot screen described.

In the screen for **inhibitors** of *PR1p::GUS* expression, seedlings were pre-incubated with the library constituents for 1 h before addition of 200 μM SA and quantifying GUS activity after 24 h as before. From the raw data it appears as if the variation of induced activity is relatively high (Figure 8A); however, the coefficient of variation (*Cv* = σ/μ) is only 0.15 when calculated across the whole screening plate, which compares favorably with the corresponding *Cv* value of 0.25 for non-induced activities (e.g., screening plate for activators, cf. Figure 7A). Irrespectively, the translational inhibitor CHX **34** was clearly identified as a strong hit, as also apparent after *Z* score transformation of the activity data, which yields a value <-2 (Figure 8B). By contrast, the mycotoxin neosolaniol **23**, which also impairs protein translation (Serrano et al., 2010), and thiomersal **37**, an antiseptic and antifungal agent, showed up as relatively weak inhibitors. This is also apparent from their *Z* scores of approximately −1 (Figure 8B). Again, the validation of such weak inhibitors would require additional experiments, such as determination of concentration dependency, bioavailability and/or stability, which is beyond the scope of this paper. The structures of all the compounds acting as activators or inhibitors of *PR1* expression identified in this small pilot screen are shown in Figure 9.

**Figure 10:**
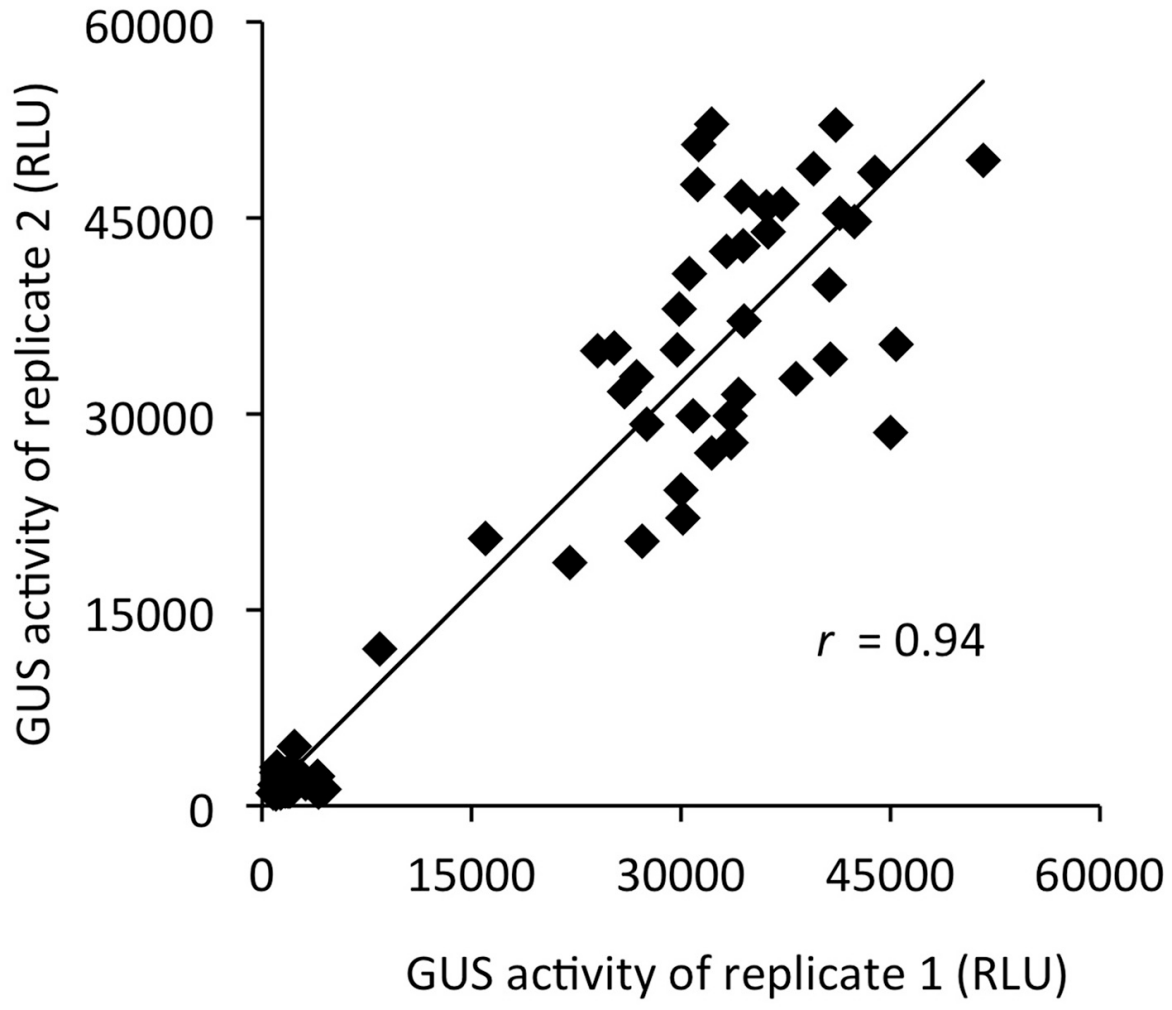
**Replicate correlation plot of screening data**. The raw activity values, replicate 1 and 2, of the two pilot screening plates for activators and inhibitors of *PR1p::GUS* expression were plotted against each other. The high value of Pearson's correlation coefficient (*r* = 0.94) indicates that the *in situ* GUS assay is robust, reliable and provides reproducible screening data.

As a final step to further characterize the outlined screening methodology, we generated a replicate correlation plot to visualize the overall reproducibility (Figure 10). The calculated Pearson's correlation coefficient (*r* = 0.94) for both primary screens is a quality metric and demonstrates a good overall reproducibility and reliability of replicates. From this we conclude that the GUS activity assay with intact seedlings provides quantitative data of sufficient robustness and accuracy to allow confident hit identification in chemical screening campaigns.

## Discussion

Here, we have established and validated a new forward chemical genetic screening method using intact *A. thaliana* seedlings harboring diverse GUS reporter constructs for direct quantification of GUS activity. Its direct application in the microplate format used for seedling growth requires only a minimum of sample handling and allows automatic acquisition of quantitative data, which are a prerequisite for unbiased identification of hits *via* numeric threshold values derived from statistical procedures (Malo et al., 2006; Birmingham et al., 2009). Clearly, this approach is superior over frequently used qualitative screening approaches that are based on visual evaluation of GUS stained tissue, which is prone to biased hit selection (Hayashi et al., 2003; Armstrong et al., 2004; Serrano et al., 2007; Gendron et al., 2008; Knoth et al., 2009; Kim et al., 2011). Likewise, the outlined procedure is superior to other quantitative GUS assays carried out *in vitro*, which rely on tissue extraction and, although accurate, are much more labor-intensive and time-consuming. The screening methodology we describe is facile, accurate, reliable, and robust and therefore suitable for high-throughput screening projects. Although this method monitors activity only *in situ* (rather than *in vivo*) it compares well with the luciferase reporter system, which allows true activity recording *in vivo* and therefore represents the most frequently used screening tool in drug discovery programs (Inglese et al., 2007). However, in plants, including *Arabidopsis*, GUS is still the prevailing reporter system in use and therefore the outlined procedure may find frequent application.

To demonstrate the reliability and robustness of the *in situ* GUS quantification with intact seedlings, we directly compared it to the conventional, frequently used quantitative *in vitro* GUS assay. Using different inducible GUS reporter lines, we observed similar patterns of substrate conversion in both assays. However, the GUS activity recorded *in situ* cannot easily be normalized to protein content or fresh weight without compromising on its ease and simplicity, but molar conversion rates can be obtained from the emitted RLU by its relation to a standard curve with known product (4-MU) concentrations. Although signal intensity is affected by seedling size, the observed variability of recorded GUS activity in replicate samples is not exceeding that of the normalized GUS activity determined *in vitro* (cf. Figures 1, 2). The same conclusion is derived from the high correlation coefficient (*r* = 0.94) of replicate samples, demonstrating high accuracy and reproducibility of GUS activity quantification. Furthermore, the *in situ* GUS assay is suitable for application to a large variety of GUS reporter lines, irrespective of their particular cell-type and organ-specific expression patterns and modes of regulation. This is not only true for the four reporter lines used in this study (Supplementary Figure 2), but also for several additional lines that we currently apply in various experiments.

To further affirm the suitability of the described GUS assay for chemical screening projects, we employed it in a small pilot screen using seedlings of the *PR1p::GUS* reporter line in search for modulators of SA signaling. Both a strong activator, ASA, and a strong inhibitor, CHX, of reporter gene expression were identified with high confidence *via* their modulation of GUS activity (Figures 7, 8). The bioactivity of both types of compound has previously been described (White, 1979; Spoel et al., 2003; Loake and Grant, 2007; Serrano et al., 2010; Meesters et al., 2014), here they served as positive and negative controls, respectively. The major advantage of the method, however, lies in the acquisition of quantitative expression data, which allows application of statistical tools for unbiased hit selection (Malo et al., 2006; Birmingham et al., 2009). In addition, quantitative screening data permit to distinguish between compounds with high and low potency, which may be useful for subsequent experimental strategies aiming at the discovery of new bioactive scaffolds. However, such weak activities as uncovered here need further critical evaluation.

In conclusion, we provided an efficient, facile, reliable and robust screening methodology, based on quantitative estimation of GUS activity in intact *Arabidopsis* seedlings, which can easily be adopted for any transgenic line harboring the GUS reporter. The acquisition of quantitative data in combination with the ease of sample and assay handling compare favorably with the convenience of truly *in vivo* activity monitoring systems such as luciferase or fluorescent proteins (GFP, RFP, etc.) and therefore the outlined methodology has great potential for broad application particularly in time- and labor-intensive large-scale chemical screening campaigns.

### Conflict of interest statement

The authors declare that the research was conducted in the absence of any commercial or financial relationships that could be construed as a potential conflict of interest.
