# Supplementary material for: Facile high-throughput forward chemical genetic screening by in situ monitoring of glucuronidase-based reporter gene expression in Arabidopsis thaliana
Source: Front Plant Sci. 2015 Jan 29;6:13. doi: 10.3389/fpls.2015.00013 (PMC4310277; doi:10.3389/fpls.2015.00013)
Supplement: Supplementary file 1 [file DataSheet1.PDF]

Supplementary Material

Facile high-throughput forward chemical genetic screening by *in situ* monitoring of glucuronidase-based reporter gene expression in *Arabidopsis thaliana*

Vivek Halder and Erich Kombrink

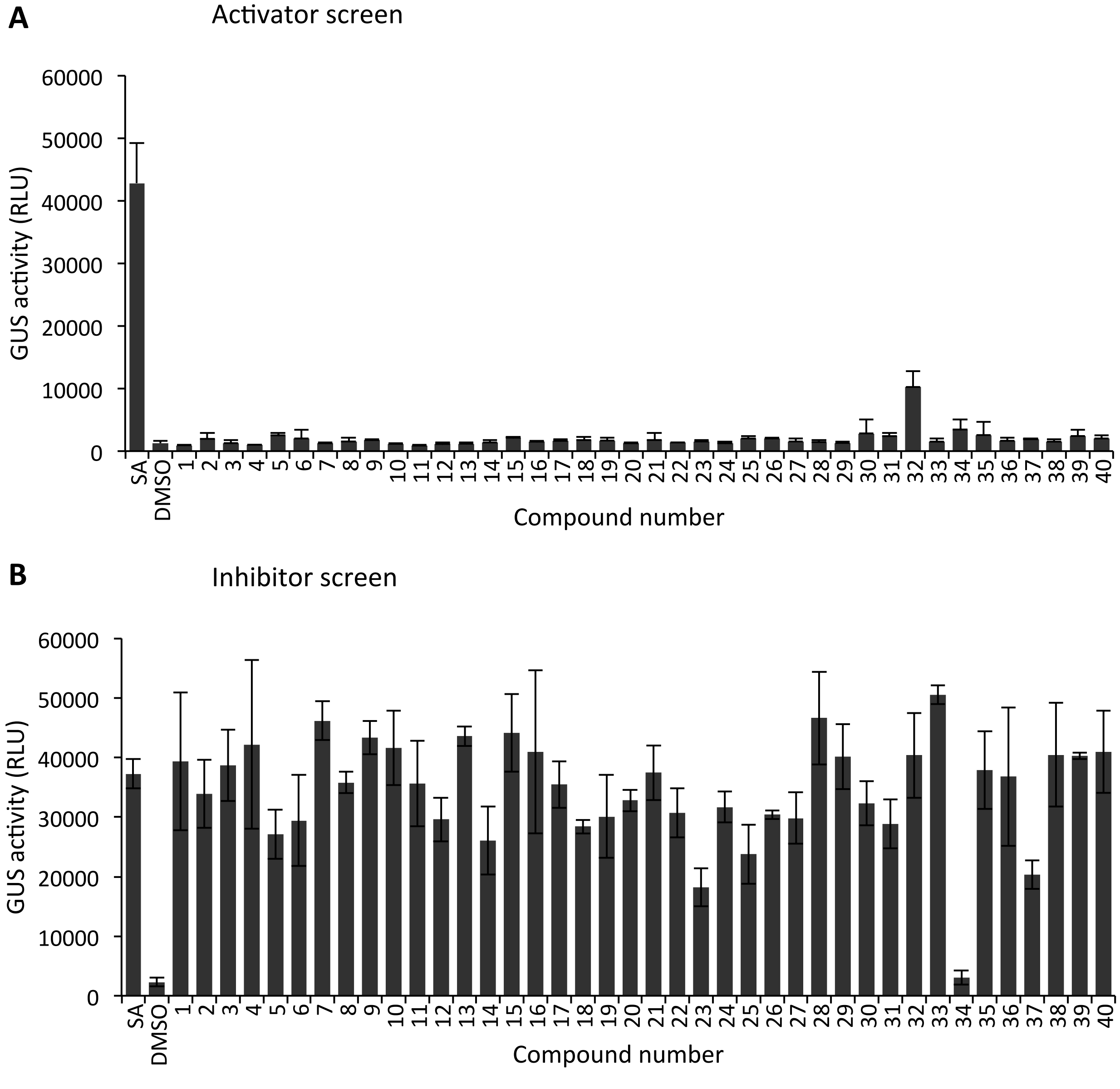

Supplementary Figure 1 | Raw screening data for activators and inhibitors of *PR1p::GUS* expression. (A) For the activator screen, *Arabidopsis* seedlings harboring the SA-responsive *PR1p::GUS* reporter gene, grown for 12 days in liquid culture, were treated with 40 diverse chemicals (20 µM) for 24 h. **(B)** For the inhibitor screen, seedlings of the same *Arabidopsis* line grown under identical conditions were treated with the same 40 diverse chemicals (20 µM) for 1 h prior to addition of SA (200 µM) to induce reporter gene expression. GUS activity of whole seedlings (*in situ*) was quantified by incubation with 4-MUG (1 mM) for 90 min. Values represent the mean activity (relative light units (RLU) per seedling) of duplicate samples that were tested for each of the 40 chemicals, the error bars indicate the corresponding high and low values. Also included with each plate are eight positive controls (200 µM SA treated) and eight negative controls (DMSO treated) and these values, in this case representing the mean (± SD), served to calculate the *Z'* factors, yielding 0.58 and 0.73 for the data sets in **(A)** and **(B)**, respectively. All values shown here were used for different normalization procedures that are shown in **Figures 7** and **8**.

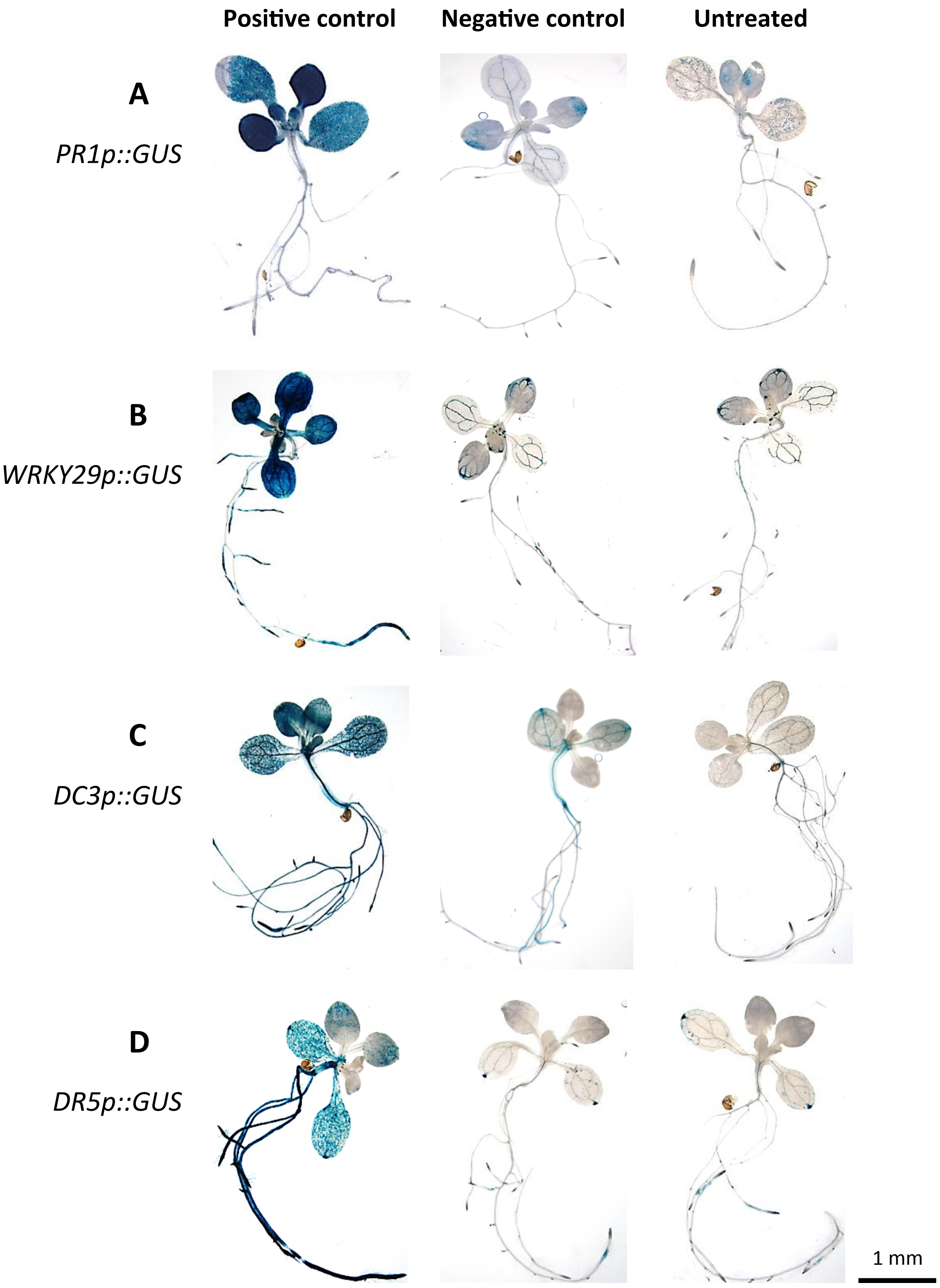

Supplementary Figure 2 | Histochemical localization of GUS expression in different *Arabidopsis* reporter lines. Seedlings of transgenic *Arabidopsis thaliana* lines harboring different inducible promoter–GUS fusions were grown for 12 days hydroponically in microplates and then treated with the respective inducer (positive control), or solvent (negative control), or left untreated for an appropriate time period to obtain high expression levels of the reporters. (**A**) *PR1p::GUS* seedlings received 200 µM SA for 24 h, (**B**) *WRKY29p::GUS* received 1 µM flg22 for 4 h, (**C**) *DC3p::GUS* received 100 µM ABA for 24 h and (**D**) *DR5p::GUS* received 5 µM IAA for 4 h. Following this treatment, the medium was removed and seedlings stained with X-gluc as described in the methods.
